# Herpes Virus Fusion and Entry: A Story with Many Characters

**DOI:** 10.3390/v4050800

**Published:** 2012-05-10

**Authors:** Roselyn J. Eisenberg, Doina Atanasiu, Tina M. Cairns, John R. Gallagher, Claude Krummenacher, Gary H. Cohen

**Affiliations:** 1 Department of Pathobiology, School of Veterinary Medicine, University of Pennsylvania, Philadelphia, PA 19104, USA; 2 School of Dental Medicine, University of Pennsylvania, 240 South 40th Street, Philadelphia, PA 19104, USA; Email: doinaa2@biochem.dental.upenn.edu (D.A.); tmcairns2@biochem.dental.upenn.edu (T.M.C.); joga@dental.upenn.edu (J.R.G.); ckrummen@dental.upenn.edu (C.K.); ghc@dental.upenn.edu (G.H.C.)

**Keywords:** glycoproteins, HSV, CMV, EBV, VZV, crystal structure, functional region, monoclonal antibody

## Abstract

Herpesviridae comprise a large family of enveloped DNA viruses all of whom employ orthologs of the same three glycoproteins, gB, gH and gL. Additionally, herpesviruses often employ accessory proteins to bind receptors and/or bind the heterodimer gH/gL or even to determine cell tropism. Sorting out how these proteins function has been resolved to a large extent by structural biology coupled with supporting biochemical and biologic evidence. Together with the G protein of vesicular stomatitis virus, gB is a charter member of the Class III fusion proteins. Unlike VSV G, gB only functions when partnered with gH/gL. However, gH/gL does not resemble any known viral fusion protein and there is evidence that its function is to upregulate the fusogenic activity of gB. In the case of herpes simplex virus, gH/gL itself is upregulated into an active state by the conformational change that occurs when gD, the receptor binding protein, binds one of its receptors. In this review we focus primarily on prototypes of the three subfamilies of herpesviruses. We will present our model for how herpes simplex virus (HSV) regulates fusion in series of highly regulated steps. Our model highlights what is known and also provides a framework to address mechanistic questions about fusion by HSV and herpesviruses in general.

## 1. Introduction

**Figure 1:**
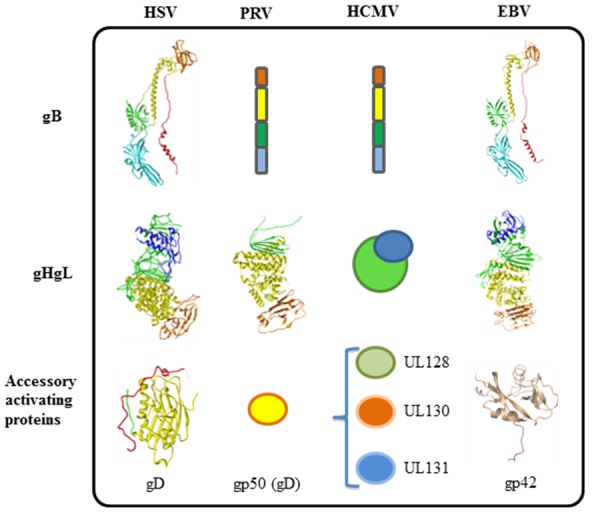
Fusion machinery of herpesviruses. All herpesviruses utilize gB and gHgL for membrane fusion. Different herpesviruses have different accessory proteins involved in regulation of membrane fusion. gD is used by alpha herpes viruses (HSV), and porcine herpesvirus (PRV) uses gp50. Beta herpesviruses (HCMV) use UL128, UL130, and UL131. Gamma herpesviruses (EBV) use accessory protein gp42. The only structures that are currently known can be accessed from the PDB database: HSV-1 gB, 2GUM; HSV-2 gHgL, 3M1C; HSV-1 gD, 2C36; EBV gB, 3FVC; EBV gHgL, 3PHF; EBV gp42, 3FD4; PRV gH, 2XQY.

Virus-cell fusion induced by enveloped viruses requires disruption of both the inner and outer layers of cellular and viral membranes. For many enveloped viruses, binding of a single surface glycoprotein to its receptor promotes pH-dependent conformational changes once within the acidic environment of an endosome, thereby bringing the viral bilayer in proximity with the host cell membrane to promote fusion. Herpesvirus entry and membrane fusion require three virion glycoproteins, gB and a gH/gL heterodimer, that function as the “core fusion machinery” [1,2,3,4,5,6,7,8,9,10] (Figure 1). 

**Figure 2:**
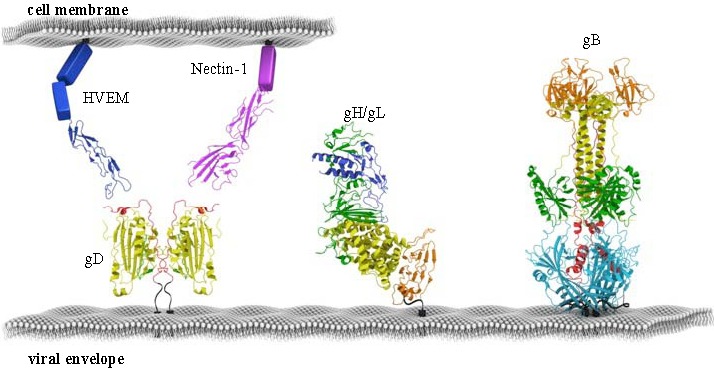
Fusion machinery of HSV. The minimal set of proteins required for HSV membrane fusion is depicted to scale. gB is structurally conserved across all herpesviruses, while gH/gL is more variable. gD is the accessory fusion protein required for membrane fusion in HSV. gD determines cell tropism by binding cellular receptors HVEM or Nectin-1. All proteins are colored according to their structural regions, as defined previously for HSV gD, HSV gH/gL, HSV gB, HVEM and Nectin-1.

A number of reports have shown that herpesviruses require additional accessory proteins to provide tropism and/or to trigger the core fusion machinery for virus entry and cell-cell fusion (Figure 1). Some examples include human cytomegalovirus (HCMV), UL128, 130 and 131 (abbreviated UL128-131) interact with gH/gL, which enables the virus to enter epithelial and endothelial cells [11] as opposed to fibroblasts but an entry receptor has not firmly been defined for CMV. In contrast, HSV can interact with two different protein receptors for entry, HVEM and nectin-1 (Figure 2) [12]. 

Both of these proteins bind to gD, the principal entry receptor of herpes simplex virus (HSV). It is of some interest that the related alphaherpesvirus, pseudorabies virus (PRV), cannot use HVEM but can use nectin-1 as a gD receptor. In the case of Epstein Barr Virus (EBV), gp42 binds to a specific cell receptor, human leucocyte antigen (HLA), which is present on B cells but not on epithelial cells, and this difference determines virus tropism [13,14]. gH/gL binds integrins alpha, beta 6 or 8 on epithelial cells as a regulator for fusion [15,16]. Another gamma herpesvirus, HHV8 interacts with the receptor for ephrine A2 to amplify signals needed for infection. There are many other examples where the entry machinery of herpes viruses up-regulate signal cascades and the following references represent a sampling: [17,18,19,20]. Our goal in this review is to emphasize our current view of the mechanistic basis for entry and cell-cell fusion by herpesviruses and how it might affect cell tropism where that has been demonstrated. Finally, we will offer our view of how HSV in particular uses four glycoproteins and a cell receptor to carry out cell-cell fusion and virus entry. 

## 2. The Two Receptors of HSV and Their Roles in Entry and *in vivo* Tropism

Proteins associated with the core fusion machinery play an important role in virus tropism, for instance through receptor selection. Clinical isolates of both HSV-1 and HSV-2 have the ability to useboth HVEM and nectin-1 as entry receptors that bind gD, regardless of what type of lesion they came from [21], suggesting that both receptors may play important roles *in vivo*. As HVEM is a TNFR-like receptor primarily found on immune cells, its role as a viral entry receptor for all strains of HSV tested remains somewhat puzzling, as T cells are not targets of productive HSV infection *in vivo*. However, the interaction may be important for modulating the host’s T-cell response to ensure survival of the virus and possibly prevent unchecked replication. For example, it has been shown that gD competes with BTLA, an HVEM ligand that reduces HVEM expression on the T-cell surface [22,23]. This competition is due to the fact that both gD and BTLA bind to most of the same residues on HVEM. Thus, HSV gD may interfere with a delicate immune balance that normally occurs in the host.

In contrast, nectin-1 is a cell adhesion molecule found abundantly on epithelial cells and at neuronal synapses [24,25], known targets for infection. Thus, its role in promoting viral replication is more apparent. Experiments using monoclonal antibodies (MAbs) to either nectin-1 or HVEM to block receptor usage in murine and human neurons showed that nectin-1 is the preferred receptor in the ultimate targets of HSV-1 *in vivo* [26,27]. Other studies to address this issue used mice knocked out for either HVEM, nectin-1, or both. When knockout mice lacking nectin-1 were infected intra-vaginally with HSV-2, the virus failed to spread to sensory ganglia [28,29]. Experiments with mice lacking either nectin-1 or HVEM showed that nectin-1 is essential for HSV-1 infection via the intracranial route and for encephalitis HVEM is largely “irrelevant”. However, recent studies showed that although nectin-1 was more important as an entry receptor into cornea, mice knocked out for both HVEM and nectin-1 were not susceptible to corneal infection by HSV-1 [30]. These results suggest that both receptors play a role in HSV entry *in vivo*, but that the balance is tilted towards nectin-1. It is interesting that at least in *in vitro* studies, nectin-1 can act as an entry receptor for PRV, bovine herpesvirus-1 (BHV-1) and B virus [31,32,33,34]. This widespread use of nectin-1 and perhaps other nectin family members may be related to how these viruses have evolved with their specific hosts. 

One exception to this widespread use of gD and nectins by alphaherpesviruses is VZV which does not have a gD gene and hence cannot use either HVEM or one of the nectins for entry. Indeed, it is still somewhat mysterious which glycoprotein functions as the receptor binding protein for VZV, although there is mounting evidence that this function is assumed by glycoprotein gE [35,36]. Furthermore, there is evidence that insulin degrading enzyme (IDE) binding to VZV gE is required for infection in an *in vitro* system, and acts as a receptor for spread of the virus from cell to cell [36,37]. However, in a SCID-human model of human pathogenesis, where human cells are grafted onto SCID mice and then infected, the experiments suggest that the interaction between gE and cellular IDE is dispensable for VZV infection of the dorsal root ganglia [38]. This illustrates how important it is to relate *in vitro* studies to the *in vivo* situation. Perhaps future experiments will identify an additional receptor that is used by VZV in both systems.

## 3. Tropism of Herpesviruses is Often Defined by Accessory Receptor Binding Proteins and Receptors

For many years, a puzzling observation about HCMV entry was that standard or laboratory strains of the virus (such as Towne or AD169) could readily infect fibroblasts but not epithelial or endothelial cells, the major target cells of this virus *in vivo*. However, sequencing of fresh clinical isolates of the virus revealed that they contains a large number of genes that were missing in the laboratory strains [39]. Among these were genes for three glycoproteins, UL128,130 and 131 (UL128-131) which are now known to be accessory proteins that bind gH/gL for infection of epithelial and endothelial cells (Figure 1) [11,40,41]. These clinical isolates were able to infect epithelial cells but not fibroblasts. In contrast, another HCMV glycoprotein, gO, which was shown to co-associate with gH/gL, was originally thought to be a gH/gL accessory protein but was more recently shown to act more like a gH/gL chaperone [42,43]. Thus, the pentameric complex defines tropism for HCMV into epithelial and endothelial cells but the presence of UL128-131 makes the virus refractory for entry into fibroblasts. In fibroblasts it appears that only gH/gL and gB are required for virus entry [44], though other accessory proteins may yet be discovered. Interestingly, only gH/gL and gB are needed for *in vitro* cell-cell fusion of a variety of cell types [8] although an unidentified cellular protein might also be involved in this process, e.g., MHC which is present on target cells [45,46,47]. On the other hand, gO promotes export of gH/gL from the endoplasmic reticulum (ER) of all three cell types and the accumulation of gH/gL in the trans-Golgi network [48]. Thus it is needed for proper insertion of gH/gL into infectious virions but apparently it is not itself incorporated into the virion. Thus, in HCMV, the presence of UL128-131 in the virion envelope is a determinant of cell tropism, though it is not clear if one of these three proteins binds a cell receptor. Indeed, several receptors have been proposed for HCMV, e.g., epidermal growth factor receptor EGFR and integrins [49]. 

In the case of HHV6, another betaherpesvirus, glycoproteins gQ1 and gQ2 bind gH/gL and promote the use of CD46 as an entry receptor on cells that express this protein [9,50,51]. Alternatively, a complex of gH/gL with another viral protein, gO, does not associate with CD46 and may allow entry into other cells that express a receptor that has not yet been defined. Again, these proteins may be determinative of which cell type is infected. The common theme is that all of these viruses employ different accessory proteins not just for receptor binding but as tropism factors. Ultrastructural studies may help clarify some of the more mysterious aspects, undoubtedly a goal of many investigators. Clearly, for beta herpesviruses, certain pieces of the entry/fusion/tropism puzzle have not yet fallen into place.

**Figure 3:**
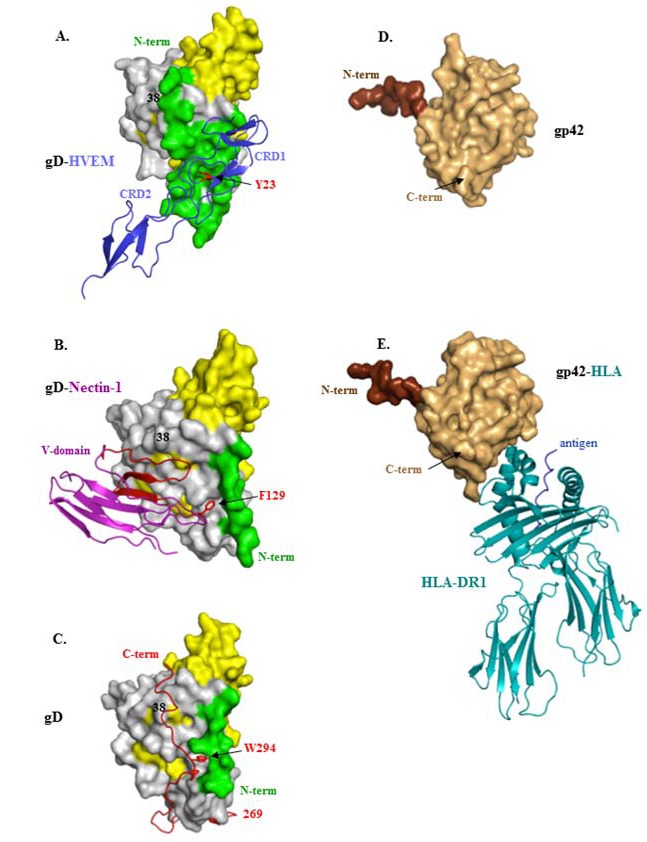
Structures of HSV gD and EBV gp42 alone or bound to their ligands. (**A**) gD(285t) bound to HVEM. The gD Ig fold is shown in yellow and its core extensions are in gray. The N-terminus (aa 1-32 in green) forms a hairpin bent at residue 21. The first two cysteine-rich domains (CRD) of HVEM are shown as blue ribbons. The essential tyrosine 23, which inserts between the strands of the gD N-terminal hairpin, is shown in red; (**B**) gD(285t) bound to nectin-1. gD is colored as in **A**. The N-terminal and C-terminal flexible residues (1-22, 260-285) are not solved. The V-domain of nectin-1 is shown as blue ribbons with beta strands CC’C” that contact gD shown in red. The essential phenylalanine 129, which inserts into a pocket near the N-terminus of gD is colored red; (**C**) Unbound HSV gD306t(307Cys) protomer. gD is colored as in **A** with the C-terminus (269-307) shown as a red ribbon. The C-terminus anchoring residue tryptophane 294 is shown in red and the position of Tyrosine 38 is indicated. The extended N-terminal 22 residues and the flexible hinge (aa. 260-268) are not visible; (**D**) Unbound EBV gp42 protein is shown in tan and residues at the N-terminus of the solved structure are colored brown (aa 83-93); (**E**) gp42 bound to human HLA protein. gp42 is colored as in **D**. The human HLA-DR1 is shown as teal ribbons and the antigenic peptide is colored blue. In all panels N and C-terminal residues of gD and gp42 are shown when visible. Representations of gD and gp42 are not drawn to the same scale.

However, what is well understood is a very elegant molecular mechanism that regulates EBV cell tropism both at the level of cell biology [14] and at the ultrastructural level (reviewed in Connolly *et al*. [4]). Although glycoprotein gp350/220 is needed for EBV attachment, it is now clear that gp42 is the entry receptor-binding protein for EBV [52]. The type II membrane protein gp42 is required for infection of B cells, but not epithelial cells [53]. When EBV infects B cells, gp42 binds the human leukocyte antigen (HLA) (Figure 3E). Importantly, expression of gp42 on the surface of the virion changes as the virus alternates between infection of epithelial cells and lymphocytes in the human host. When the virus is produced in epithelial cells, the virion contains abundant gp42, which allows efficient infection of B cells. Conversely, when the virus is produced in B cells, the amount of gp42 in the virion is reduced, possibly due to being bound to the receptor (HLA). B cells also make a truncated soluble form of gp42 that appears to inhibit entry of the virus into epithelial cells. Indeed, EBV fusion with an epithelial cell is initiated by an interaction between several integrins and gH/gL [15,54] and gp42 blocks binding of gH/gL to integrins [5,16,55]. At the same time, in epithelial cells less gp42 is degraded and the resulting virions have a high gp42 content that targets these virions to B-lymphocytes [56]. It is of interest that gH/gL itself appears to play a crucial role in defining cell tropism. It was recently reported that a KGD motif of EBV gH/gL may play a role in infection of both B cells and epithelial cells. The authors feel that this acts as a tropism switch [16], although this is still under debate by others in the EBV field.

Structural analyses and mutagenesis of unbound gp42 and gp42 bound to HLA indicate a conformational change [57,58,59]. Because gp42 also binds specifically to gH/gL, it is possible that conformational changes that occur when gp42 binds its receptor help position it to bind its gH/gL partner [57,58,60,61]. For instance, gp42 contains a hydrophobic pocket that widens in response to HLA binding and it has also been suggested that this widened pocked is “reserved” for a second high affinity gH/gL binding site. 

## 4. Structural Studies of HSV gD and Its Two Protein Receptors

A major development in our understanding of how HSV gD interacts with its receptors was the determination of the structures of gD bound to HVEM and more recently to nectin-1 [62,63,64,65] (Figure 2 and Figure 3A–C). The form of gD used for crystallization of both complexes was truncated at amino acid 285 because of its very high affinity for gD compared with that of longer forms [66,67,68]. In the gD/HVEM complex (Figure 3A), gD consists of a V-like immunoglobulin (IgV) core that acts as a scaffold for N- and C-terminal extensions. In this complex, the N-terminus of gD forms a hairpin structure into which Tyr23 of HVEM inserts, thereby stabilizing the interface [62]. An intermolecular β-sheet between gD and the first cysteine-rich domain of HVEM as well as key hydrogen bonds between gD and the second cysteine-rich domain of HVEM are also important. Mutational analysis of the interface confirmed that Tyr23 of HVEM and gD residues that form the beta sheet with HVEM are critical to maintain the integrity of the complex [69,70]. When gD is not liganded to HVEM, the first 21 amino acids are flexible and some are even too disordered to be resolved (Figure 3A) [62]. Thus, HVEM contributes to formation of the more structured hairpin loop at the N-terminus of gD that is critical for fusion and entry of HSV into cells bearing HVEM [62,63,71]. This conformational change raised the possibility that it accounted for how binding of gD to either receptor might trigger downstream events, assuming that such a change would be common to binding of both receptors. 

Therefore, we and others mapped the binding of nectin-1 on gD (and *vice versa*). The initial studies, done both by mutagenesis and epitope mapping, showed that all of the residues of nectin-1 involved in gD binding reside in the distal V-domain of this Ig-like structure [72,73,74]. When expressed alone, this domain binds to gD with the same affinity as the full nectin-1 ectodomain [68]. Specific residues within the V-domain that contribute to binding of gD were also identified [75,76,77,78,79]. Surprisingly, the site on gD for nectin-1 binding was mapped to residues mostly downstream of the N-terminus that binds HVEM (Figure 3B) [76,77,80,81,82] and suggested that the N-terminus of gD was not involved in nectin-1 binding. To test this possibility, a mutant was constructed to lock the N-terminal residues of gD into a hairpin with a disulfide bond between amino acids 3 and 38 (Figure 3C). The gD mutant A3C-Y38C was still able to bind HVEM but was unable to bind nectin-1 [77], suggesting that: (1) the gD hairpin could be pre-formed and still allow HVEM binding; and (2) binding nectin-1 required a flexible N-terminus of gD. Thus, these data showed that there was no conformational change at the N-terminus of gD that was common to binding of gD285 to these two receptors. 

Once the structure of gD285 bound to nectin-1 was finally solved [64,65], it was confirmed that gD binds strictly to the V-domain of nectin-1, the same domain used for trans-interactions of nectin-1 with itself and other nectins [64,65,83] (Figure 3B). The crystal structure of the gD/nectin-1 complex confirmed and expanded the results of all of the previous mutagenesis experiments and also pointed to a critical role for Phe129 of nectin-1 in maintaining the interface with gD but protruding into a pocket within the IgV-like core of gD (Figure 3B). 

Since resolution of the gD285/HVEM and gD285/nectin-1 structures did not reveal a common conformational change in gD, the connection between receptor binding and activation of fusion remained unanswered*.* However, gD285 is truncated well short of the transmembrane region (TMR) at 316. It was used for crystallization due to its high affinity (10^−8^ M) for both receptors which allowed stable complexes to form. In contrast, a longer form, gD306 has only micromolar affinity for HVEM or nectin-1 [66,67,68]. This contrast in affinities suggested that residues 285-306 impeded but did not prevent receptor binding. Interestingly, truncations as short as gD260 also had higher affinity for receptors than did gD306, again due to a higher rate of complex formation. Studies using the various truncated forms of gD to trigger entry of a gD-null virus led to the conclusion that residues 260-285 constitute a “profusion domain” [84]. A confounding observation was that consecutive 10 amino acid deletions along this region did not disrupt fusion [81]. However each 10 amino acid deletion left a large portion of this domain intact. Thus, it was possible that none of these deletions affected the overall position of this portion of gD. It was hypothesized then that HVEM or nectin-1 binding to gD306 required a conformational change to move the impediment caused by the C-terminal residues. Since these residues are missing in gD285, no conformational change would be needed for receptors to bind. 

To test this idea it was essential that a method be applied to stabilize the flexible C-terminus so as to reveal the residues between amino acid 260 and 306. For this, a mutant with a cysteine residue right after residue 306 was constructed (gD306t_307Cys_), forming a dimer locked at the C-terminus. The hope was that the added cysteine would result in an intermolecular disulfide bond between two monomers of gD thereby locking the two C-termini and the residues proximal to it (Figure 3C shows the protomer and Figure 2 shows the dimer) [63]. Indeed, this form of gD was crystallized and revealed that the positions of residues 269-306 snake closely across the gD core so that the C-terminus is in fact close to the N-terminus of each monomer. In this structure the position of residues 269-306 prevents formation of the hairpin loop needed for HVEM binding to gD and obscures residues needed for nectin-1 binding to gD. In fact, gDt306_307Cys_ was unable to bind either receptor. According to this structure, these C-terminal residues must move away to allow both HVEM and nectin-1 to bind for a common conformational change to occur [85]. 

To confirm the validity of this structure, five additional disulfide bonded forms of monomeric gD were made choosing amino acids close enough in the structure of gDt306_307Cys_ to restrain movement of various portions the C-terminus [86]. Mutant 1 was the least restrained (locking amino acid 242 to 274) and mutant 6, the original one described above was the most restrained, locking amino acid 37 to amino acid 302 in the context of a gD dimer [86]. Mutants 4, 5 and 6 were unable to bind receptors or trigger fusion and were therefore uninformative. Notably, mutants 1-3 retained the ability to bind receptors but were impaired in their ability to mediate fusion, with mutants 1 and 3 being partially crippled and mutant 2 (K190C-A277C) being completely unable to trigger either cell-cell fusion or to complement a gD-null virus. Thus, mutant 2 clearly separated the ability of gD to bind receptor from its ability to promote fusion. These results suggest that this second post-binding function of gD required only a portion of the C-terminus to be flexible and that residues in WT gD that directly interact with gH/gL and/or gB may also be obscured by the ectodomain C-terminus, and are then revealed as the C-terminus moves away from the core of gD. 

Interestingly, the structure of gDt306_307Cys_ shows that the C-terminal residues of the gD ectodomain are stabilized by the side chain of Trp294, which, along with Pro291 fit into a pocket formed by the N-terminus and the central helix of gD (Figure 3A,B). This is the same pocket that is stabilized by Phe129 of nectin-1 at the gD/nectin-1 interface. In unliganded gD (Figure 3C), this structure of gDt_307Cys_ predicted that Trp294 of gD has to be displaced in order for nectin-1 to stably form a complex with gD. In fact, mutation of Phe129 of nectin-1 to either Leu or Ala has a negative effect on gD affinity for nectin-1 [87]. Mutation of Trp294 of gD increases binding of gD to nectin-1 but ablates the ability of a virus with this mutation to complement a gD null virus [63]. Interestingly, the fact that single amino acids such as Phe129 of nectin-1, Tryp294 of gD and Tyr23 of HVEM all act as “hot spot” residues that hold the interface between two proteins together has been documented for other proteins [88].

All of these mutants confirmed the structure of the C-terminal residues of the ectodomain of gD. Importantly, a comparison between unliganded gD, as seen in gD306t_307Cys_ (Figure 3C), with gD bound to either receptor shows that C-terminal residues of the gD ectodomain must be displaced to allow either receptor to bind (Figure 3A,B). This common conformational change led to a model for receptor-activated triggering of the fusion machinery by gD [63,86]. The model proposes that the gD ectodomain in the virus assumes a similar conformation as seen in the structure of gDt_307Cys_. In support of this a monoclonal antibody (AP7) originally isolated by Minson and colleagues [89] binds to an epitope that includes both the N- and C-termini of the gD ectodomain [90]. This model also suggests that receptor binding sites are normally hidden in virion gD and are revealed only when the virus comes in contact with the appropriate receptor on a target cell.

Conformational changes have also been noted between the unbound and HLA Class II-bound forms of gp42 of EBV (Figure 3D,E), though they appear not to be quite as dramatic as the ones seen between gD and its receptors [4]. We suggest that this mechanism works to the advantage of both herpesviruses by preventing premature activation of the fusion machinery before the reaches its appropriate target cell within its human host. 

## 5. Virus Neutralizing Antibodies to gD Target Its Two Separate Functions

**Figure 4:**
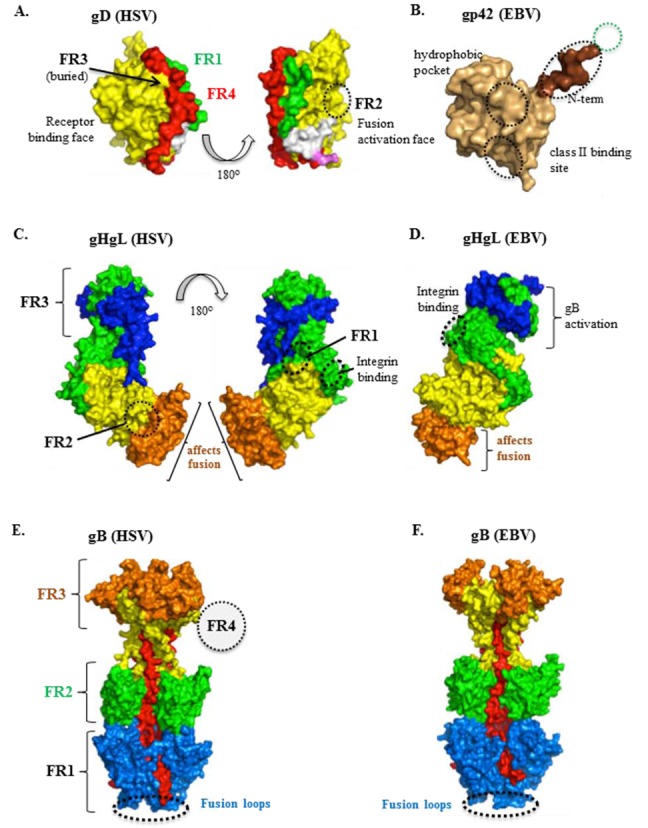
Functional regions in herpes glycoproteins. All proteins are colored according to their structural regions, as defined previously for HSV gD, HSV gH/gL, and HSV gB. (**A**) HSV gD. Two views of the monomeric, unbound form of gD are shown. The green (part of which is FR1; 27-43) represents the N-terminus which forms the HVEM-binding hairpin in the gD-HVEM complex. The Ig-like core (contains FR2, residues 125-161, and FR3, residues 225-246) is shown in yellow and the C-terminus (contains FR4; 277-310) in red. For reference, the MC2 (234-250; white) and MC10 (262-272; pink) epitopes are shown; (**B**) EBV gp42. Defined regions are as described in Kirschner *et al*. Binding sites for proteins gH/gL (residues 36-81, within the green dotted circle) and HLA class II are indicated, as well as a distinct hydrophobic pocket essential for membrane fusion (black circles). The last two regions were defined by linker insertions; (**C**) HSV gH/gL. Two views of gH/gL are shown. The gH structural domains are H1 (green), H2 (yellow), and H3 (orange). gL is shown in blue. HSV gH/gL FRs are defined by the epitopes of neutralizing MAbs: LP11 (FR1), 52S (FR2), and CHL17/32 (FR3). The integrin binding site (residues 176-178) is also indicated. Numerous mutations that affect cell-cell fusion map to domain H3; it is currently unknown if this is a functional domain separate from FR2 (which spans H2-H3); no known neutralizing MAbs map here; (**D**) EBV gH/gL. gL (blue) contains a hypothesized site of gB interaction. The integrin binding site (residues 188-190) is also indicated. Numerous mutations that affect fusion map to domain H3, as well as the epitope of the neutralizing MAb CL 59; (**E**) HSV gB. HSV gB FRs are defined by the epitopes of neutralizing MAbs: SS55/SS106/SS144 (FR1, blue and red, fusion domain), C226/H1838/H1781 (FR2, green, gH/gL interaction domain), SS10/SS67-69 (FR3, orange, receptor-binding domain). The epitope of MAb H1817 (FR4, dotted circle) maps to the unresolved N-terminus. The fusion loops are indicated at the bottom of the structure; (**F**) EBV gB. Chimeric EBV mutants have suggested that structural domains III (yellow), IV (orange) and V (red) are important for gB-gH/gL binding. The fusion loops are indicated at the bottom of the structure.

Previously, we and others identified a number of neutralizing monoclonal antibodies (MAbs) directed at HSV gD [91,92]. Many of them blocked binding of gD to one or both of its protein receptors [93,94]. In addition linker insertion mutants defined four functional regions (FR) of gD (Figure 4A and [90]). Forms of gD with mutations in each of these regions ablated or reduced virus infection, while having little or no effect on MAb binding. It is interesting that all of the epitopes for these neutralizing MAbs are found in FR1 and FR3 (Figure 4A), as originally defined by Chiang *et al*. [90]. We recently discovered an antibody, MC2, that neutralizes virus but does not block receptor binding and instead actually enhances it [95]. A portion of the epitope for this conformation-dependent MAb may structurally overlap FR2 on a different face of gD than the face that binds other neutralizing MAbs and the receptors (Figure 4A) [95]. Thus, MC2 fits the model of a neutralizing antibody that blocks the fusion-activating function of gD while at the same time enhances receptor binding, possibly by moving the C-terminus away from the receptor-binding sites. Interestingly, a number of non-neutralizing MAbs whose epitopes are along the C-terminus of gD (called FR4 in Figure 4A) also enhance receptor binding, suggesting that all of these MAbs induce the C-terminus to be in an “open” configuration. The unique properties of MC2 are likely conferred by the location of its epitope, such that antibody binding alters gD conformation to promote receptor binding. At the same time, antibody binding to the MC2 epitope interferes with conformational changes or interactions required for fusion. We hypothesized that residues either within or near the MC2 epitope might play a role in the second function of gD. In this way MC2 is analogous to cysteine mutant 2 in defining the second gD function. Interestingly, both the neutralizing capacity and rate of neutralization of virus by MC2 is enhanced when combined with several of the non-neutralizing MAbs, all of which map to the same linear epitope (amino acids 262-272). We envision that stimulation of non-neutralizing antibodies by vaccines against HSV may play a role in protection by enhancing the neutralizing activity of specific antibodies against gD and perhaps other glycoproteins. A question that remains to be answered for MC2 is what step in the pathway to fusion does it block? For EBV, accessory proteins such as gp42 bind to gH/gL once it binds its receptor. Perhaps receptor-activated gD also binds gH/gL, although no data have been reported that soluble, purified gD binds directly and tightly to purified gH/gL. Such an interaction has been reported to occur in bimolecular complementation studies [96] and in cell extracts [97]. Other evidence that such an interaction occurs will be described in a later section of this review. 

## 6. Structure-Function Analysis of gH/gL

The heterodimeric complex gH/gL is a component of the core fusion machinery of all herpesviruses. gH/gL is also a major target of HSV neutralizing antibodies, as first shown by Peng *et al*. [98] and more recently for HCMV by Macagno *et al*. [99], highlighting its importance for virus infection. HSV-1 gH is an 838 amino acid glycoprotein with a large ectodomain and a single C-terminal transmembrane anchor [100]; gL is a 224 amino acid glycoprotein lacking a transmembrane region. Both proteins have signal peptides directing them into the secretory pathway. In HSV-infected cells and on mature virions, gH and gL are always found together in a stable 1:1 complex [98], as gL is required for correct folding and trafficking of gH. Specifically, expression of HSV gH in the absenceof gL results in its intracellular retention, presumably in the endoplasmic reticulum. This abrogates generation of important epitopes [101,102,103] and prevents its incorporation into the viral envelope [104]. When HSV gL is expressed in the absence of gH, some is secreted from mammalian cells [105,106] although a substantial amount is retained inside the cells in its immature form (unpublished observations and [105]). 

Until recently, little was known about the role of gH/gL in fusion. The ability of transiently expressed Kaposi’s sarcoma-associated herpesvirus (KSHV) gH/gL to mediate low level syncytia formation in the absence of any other viral proteins [107] suggested it may have some inherent fusogenic properties. Although similar observations have been made with gH/gL from HCMV and varicella zoster virus (VZV) [35,108], not all gH/gL proteins have such properties. In one study it was claimed that HSV gH/gL was responsible for hemifusion, whereas gB was involved with full fusion with a target membrane [109]. However, when the essential experiments of this study were recapitulated by a second group, they could not detect gH/gL hemifusion [110].

Several reports suggested that gH of a variety of herpesviruses functions as a viral fusion protein [35,107,108,109,111]. gH has been likened to class I viral fusion proteins based on studies carried out with peptides corresponding to predicted heptad repeats and fusion peptides [112,113,114,115], hydrophobic stretches within viral fusion proteins capable of binding to membranes. Several of the proposed fusion peptides of gH induced fusion in liposomes as synthetic peptides [114,115,116] but the full-length protein and the soluble, truncated forms of gH/gL do not associate with lipid bilayers [117,118].

Recently, the crystal structure of nearly the entire gH ectodomain bound to full-length gL from HSV-2 was determined, revealing a boot-shaped heterodimer approximately 80Å X 70Å (Figure 1 and Figure 2) [119]. Shortly after this structure was reported, two additional reports revealed the structure of EBV gH/gL and a partial structure PRV gH [120,121] (Figure 1). In the case of HSV-2 gH/gL, the C-terminus of the gH ectodomain, which would normally lead into its transmembrane region, is located near the “sole” side of the “toe”. The unusual shape of the gH/gL structure is reminiscent of the 15–20 nm glycoprotein spikes, observed by cryo-electron tomography, which emerge from the HSV-1 envelope at an angle and often appear curved [122]. Therefore, each gH/gL molecule on the virion surface probably appears to be standing on its “toe” and leaning to the side (Figure 2). Unexpectedly, the complex revealed by the crystal structure does not resemble any known viral fusion protein. In fact, unlike any viral fusion protein described to date, gH/gL does not form a trimer [123,124,125]. In the case of EBV gH/gL, the complex is more cylindrical than boot-shaped (Figure 4D) [121] but the essential domain structure is quite similar.

HSV-2 gH has three structural domains: an N-terminal domain that binds gL (H1, colored green in Figure 4C), a central helical domain (H2, colored yellow in Figure 4C), and a C-terminal β-sandwich domain (H3, colored orange in Figure 4C). For clarity, throughout this review we will refer to gH structural domains in EBV and PRV using these designations as well. The N-terminal domain of gH lacks a stable folded core and is stabilized by gL (colored blue in Figure 4C), which wraps around it, forming a clamp (Figure 4C). The second and third domains of gH of HSV, EBV and PRV are highly conserved in structure (Figure 1 and Figure 4) and in fact these regions are well conserved in sequence across herpesviruses in general. Their hydrophobic nature has led investigators to suggest they play a supporting but key role in fusion (reviewed in [4]). However, in the crystal structure, peptides predicted to be fusion peptides based on their hydrophobicity are buried helices or β hairpins in these C-terminal domains of gH. In fact there are no gH peptides that are structurally similar to known fusion peptides [119]. Additionally, predicted heptad repeats (often found in fusion proteins) [113,116,126] actually form helices, likely precluding a role in fusion. Thus, solution of the structure of gH/gL contradicts previous assumptions that this heterodimer is a fusion protein that works in tandem with gB. Instead, it provides an intriguing new puzzle as to how it is utilized to complement or regulate the fusion activity of gB. 

Although gH proteins are conserved among different herpesviruses, the sequence conservation is uneven across the protein [119,127]. Domain H1 is the most divergent, while domains H2 and H3 are more conserved, with each of the latter two domains having the same fold in HSV-2, EBV, and PRV gH (Figure 2 and Figure 4D). Yet, in EBV, the overall gH/gL structure is more of an elongated cylinder than a boot (Figure 4D) [121]. The strong conservation of domain H3 suggests that it is functionally important. In support, several non-functional mutations map to this domain in both HSV-1 [128] and EBV [129] and a potent neutralizing MAb (CL 59) for EBV is directed at this domain [129]. Since this is the most conserved domain of gH, one can speculate that H3 serves a similar function in other herpesviruses. It is noteworthy that the N-terminal region of gH that binds gL as well as gL itself is strikingly different in the EBV and HSV proteins. Indeed, gL proteins are non-conserved in sequence across the herpesviruses. Thus, it has been argued that gL has evolved in tandem with changes to the structure of H1 [4,119]. An *in silico* model of the gH/gL structure of VZV has been calculated by Arvin and colleagues [130] and bears similarities to its HSV orthologs, suggesting that the gH/gL structures of herpes viruses in general will bear similarities to those that have already been solved by X-ray crystallography.

Neutralizing MAbs directed at HSV gH, gL, or both have been useful in defining functional regions (FR) of the heterodimer (Figure 4C). Presumably, each of these regions plays a different role in gH/gL function, although additional data will be needed to support this idea. For HSV gH/gL, we define FR1 by the mapping studies done for the LP11 epitope, since it is the most potent neutralizing antibody yet isolated against this protein. Its residues were defined by monoclonal antibody-resistant mutants and map to different places in the N-terminal half of gH [119,128,131]. In addition to its effect on virus replication, MAb LP11 blocks cell-cell fusion and prohibits the interaction of gB with gH/gL, as seen by bimolecular complementation [119]. We therefore think that FR1 is the site of binding of gH/gL to gB, a necessary interaction for fusion [119]. Interestingly, the same face of gH/gL is implicated for gB binding in EBV, albeit slightly further upstream on the heterodimer, within gL [132,133] (Figure 4D). FR2 of HSV gH/gL is defined by the position of the 52S epitope [131] and is on the opposite face of gH/gL when compared with the position of the LP11 epitope. In addition to being a neutralizing antibody, 52S inhibits cell-cell fusion. Importantly it does not block the association of gH/gL with gB as seen by bimolecular complementation [119]. We believe this interaction is necessary for and precedes cell-cell fusion [134]. These results suggest that FR2 carries out a function that occurs after that carried out by FR1. Lastly, FR3 includes the epitopes of MAbs CHL17 and CHL32, located at the gH N-terminus (residues 19-38) [103,135]. These two MAbs not only neutralize virus and block cell-cell fusion, but also limit cell-cell spread. Three other gH/gL MAbs also inhibit spread: CHL2 (whose epitope is in domain H1, near gH residue 116), and CHL39 and CHL18 (whose epitopes are within the gL C-terminus) [103]. Thus, at this time we define FR3 to include both H1 and the gL C-terminus (Figure 4C). FR3 is unique from FR1 and FR2 in that it includes MAbs that affect cell-cell spread. Unfortunately, there are no comparable MAbs for EBV and this portion of the structure is missing from that which was solved for PRV gH/gL [120].

Although its role as part of the core fusion machinery is still open to debate, the requirement of gH/gL for fusion is undisputed. Important clues about how gH/gL functions may lie in its close association with accessory proteins, including UL128-131 of CMV [40] and gp42 (reviewed in [4]). In HSV, bimolecular complementation was used to show an association between gD and gH/gL [96,136]. HSV gH/gL also contains an integrin binding motif (RGD) (Figure 4C,D). However mutation of RGD to RGE had no effect on the ability of the recombinant virus to replicate [128]. There is conflicting evidence as to whether or not integrins are necessary for HSV gH/gL to bind to cells [137,138] but a similar site has been found in EBV gH/gL [15,121]; their positions are shown in Figure 4C for HSV and Figure 4D for EBV. Although the role of integrins and gH/gL function has not yet been fully resolved, the fact that the integrin motif can be mutated without affecting virus infection suggests that gH/gL binding to cellular integrins does not trigger fusion. 

Based on the unique structure of gH/gL resolved in whole or in part for several herpesviruses, we propose that it is not a fusion protein but instead its function is to activate the inherent fusogenic capability of gB. As such, gH/gL may provide a signal to gB that is functionally analogous to the triggering of fusion proteins of RNA viruses by low pH [123]. Indeed, conversion of the gB analog VSV G from a pre- to a post-fusion state depends on the acid environment of the endosome that VSV is taken into [139]. Other fusion triggering mechanisms such as protease cleavage, e.g., Nipah virus [140,141], may also be functionally analogous to the action of gH/gL. 

## 7. The Structure of gB and Its Role in HSV Entry

Although early observations of gB mutants with hyperfusogenic phenotypes suggested the involvement of gB in fusion [142], the crystal structure provides the most convincing evidence that gB functions as a viral fusion protein [143]. Indeed, its domain structure is quite similar to that of G, the fusion glycoprotein of vesicular stomatitis virus (VSV) [144] and the fusion protein of baculovirus gp64 [145]. As these proteins show no sequence similarities, the similarity in structure is remarkable given that these proteins belong to unrelated viruses. Because these proteins bear structural features of both Class I and Class II fusion proteins, they now constitute a new class of fusion proteins, Class III. In each case, the Class III proteins are trimeric with three long central α-helices (like Class I fusion proteins) and what appear to be two internal fusion loops per protomer, reminiscent of the single fusion loop of Class II fusion proteins [123]. 

In the structure, the gB ectodomain trimer forms a spike-like molecule with the approximate dimensions of 85Åx80Åx160Å. Similarly shaped spikes of gB have been observed on HSV-1 virions using electron microscopy [146]. Each protomer consists of five distinct structural domains in which domain I is located at the base of the gB spike, proximal to the membrane. Moreover, domain I contains two proposed fusion loops, as revealed by a series of fusion-loop mutants of both HSV and EBV [147,148,149]. The two loops constitute a fusion domain on each protomer such that hydrophobic residues are flanked by charged residues [149], and mutational analysis provides good evidence that the proposed fusion loops of gB are in fact responsible for membrane insertion. Among the mutants constructed, three with a null phenotypes for cell-cell fusion showed major defects in egress [150] as well as viral entry [149], indicating that fusion events associated with both entry and egress of HSV require and use gB in the same fashion. 

## 8. Understanding the Conversion of Pre-Fusion Form of gB to a Post-Fusion State is Hampered by the Lack of a Pre-Fusion Structure

Unlike gB, there are now structural data for both pre-fusion and post-fusion forms of VSV G. The structure of the gB ectodomain has obvious similarities with the extended post-fusion but not the folded-in pre-fusion structure of VSV G [144,151]. It is therefore likely that the available structure of gB represents the post-fusion conformation, but this is yet to be confirmed. Conformational changes to gB in response to low pH, as in endosomes, have been reported [118,152], although crystal structures of truncated gB (gB730) at low pH suggests that many of these reported changes are relatively small [153] and do not resemble the profound changes in structure seen for VSV G [151]. Of note however, in the structure of gB730, the fusion loops are positioned quite close to where the transmembrane region (TMR) would begin. There is also a membrane proximal region from amino acid 730-773 (just upstream of the TMR) for which structure has not been resolved. This region is essential and it is not clear where these residues are in a pre- or post-fusion form of gB. A model for such a form was suggested for EBV gB, based on that of VSV G [154]. This model suggests that conversion of gB from pre- to post-fusion involves the same “foldback” mechanism seen for most if not all fusion proteins of RNA viruses [123]. However, since gB cannot function as a fusion protein in the absence of gH/gL it may be synthesized or processed into a novel form. Two possibilities that have been explored are the role of pH and proteases in gB activation. Although some gB proteins, e.g., gB of EBV undergo a protease cleavage that may be involved in its activation [155], there is no evidence that this occurs with HSV gB. Furthermore, mutation of the protease cleavage site in bovine herpes virus type 1 (BHV-1) and PRV gB has no effect on virus infectivity [156], although more subtle effects on cell-cell fusion may occur. Thus there may be some variability in how gB reaches a fusion active state and pH may play a role in HSV fusion when it occurs in endosomes [152]. Understanding the structural basis for the transition of gB from a pre- to post-fusion state and how it is triggered remain one of the challenges for the future. 

## 9. Functional Studies of gB with Mutants and Monoclonal Antibodies

Until the structure of gB was solved, studies of constructed mutations was a frustrating exercise, which is unsurprising given the complex structure of the trimer [157,158,159,160]. Once the structure was solved, the previously constructed mutants could be mapped onto the gB structure [160,161,162] and new mutagenesis studies could be carried out in a more rational and targeted fashion, e.g., studies of the fusion loops [148,149,163,164]. 

Because gB is the target of many neutralizing antibodies, investigators have used MAbs to map important regions of gB [134,149,160,165,166,167,168]. Once the structure of HSV-1 gB was solved, epitope mapping efforts were directed at defining functional regions (FR) on the structure itself. Dividing gB into FRs based on where epitopes for neutralizing MAbs map has pinpointed places that must be involved in gB function. Based on this mapping, 4 FRs have been proposed for HSV (Figure 4E and [168]). FR1 contains the fusion loops [148], FR2 is located on the outward-facing middle region of gB, while FR3 maps to the outer ridge of the gB “crown” [143]. Finally, FR4 is composed of a region at the N-terminus of gB which was not solved in the crystal structure. In addition to being the site of binding of several neutralizing MAbs, it contains the heparan sulfate binding region [159] and might also bind to other cell surface molecules [149,168,169,170,171]. Both FR1 and FR2 contain Pleckstrin-homology domains (PH) suggestive of interactions with both lipids and proteins [143]. The location of one of these domains in FR1 is consistent with the fact that gB inserts into liposomes via its fusion loops. Location of the other PH domain in FR2 suggests that this region may be involved in the interaction of gB with gH/gL. Evidence for this will be summarized in section 12 below. It should be noted that EBV may have similar functional regions but no mapping with MAbs has been reported thus far.

## 10. Interactions Between gB and gH/gL

Since gB and gH/gL are both needed for fusion, a major puzzle in the field has been to decipher whether they work sequentially or as a complex that could potentially also include gD and receptors. Earlier, it was suggested that entry occurs in a stepwise fashion beginning with gD, followed at some point by gH/gL [172]. However, complexes of HSV gB-gH/gL have been detected in lysates of infected and transfected cells [97,173]. Complexes between CMV gH/gL and gB can also be immunoprecipitated from lysates of cells transduced with adenovirus vectors carrying genes for gB and gH/gL [44], but even so, it does not mean that this complex represents a functional unit for fusion in intact cells. A more biologically relevant approach is one that would occur in intact cells, especially under conditions leading to fusion. 

Recently, bimolecular fluorescence complementation (BiMC or BiFC) was developed to detect protein interactions in intact transfected cells [174,175,176,177] using confocal microscopy. In the first reports applying this technique to HSV glycoproteins, we and others found that gB and gH/gL can interact with each other, but only when both gD and a gD receptor are also present [96,136]. However, in a follow-up study, it was reported that gB interacts with gH/gL in the absence of fusion or gD [173]. This would agree with the *in vitro* work, but would also mean that the interaction was not driven by gD binding to receptors. However, the latter study was flawed by overexpressing a mutant form of gB trafficked in an abnormal manner. As pointed out by the developers of the assay [175,176,178,179] it is essential that the proteins not be over-expressed and that they be expressed in the proper cell compartment to avoid artifacts. 

To gain more evidence that the interaction between gB and gH/gL only occurs in response to binding of gD to one of its receptors and is an essential step prior to fusion, MAbs to gB and gH/gL were used to try to block the interaction between gB and gH/gL [119,134]. The rationale was that if the interaction is essential for fusion/entry, it should be blocked only by antibodies that neutralize HSV infectivity or by antibodies that block fusion. Likewise, non-neutralizing MAbs should have no effect. Moreover, MAbs that blocked BiMC but not fusion would separate sites required for gB-gH/gL interactions early in fusion from those required at later stages. Indeed, most of the neutralizing MAbs were able to block fusion and a subset of antibodies to gB, as well as one to gH/gL, only blocked the interaction with gH/gL, but not fusion. Among the former, the epitopes for many of them mapped to a region that contains the fusion loops of gB [148,149].

Among the latter group of MAbs were several that blocked fusion but not interaction with gH/gL, and mapped to a region not resolved in the crystal structure near the N-terminus of gB, suggesting that this region has an as yet unknown function, perhaps involving interaction with a cell protein [168,169]. Fewer neutralizing MAbs were available for gH/gL but one in particular, LP11 [131,180], was able to block both fusion and the interaction with gB [119]. Additional MAbs with known epitopes together with mutants will eventually pinpoint the site of the interaction between gB and gH/gL. Of course, the best way to detail the interaction will be to co-crystallize gB with gH/gL, a challenge for the future. Together these studies suggest a stepwise progression of events from binding of gD to receptor to the end result of fusion. One theory regarding how gH/gL functions in fusion was suggested for PRV [120]. This theory focuses on an extended “flap” of gH that masks a conserved hydrophobic patch in the most C-terminal domain of this protein. It suggested that unmasking of this flap exposes a hydrophobic surface that then participates in fusion. Since this region of gH is the most highly conserved among herpesviruses, this mechanism might be broadly used. Indeed, there are several publications supporting the idea that this region of the ectodomain, as well as the transmembrane region may impact fusion [181,182,183].

## 11. Fusion *in trans* Reveals Additional Information about gH/gL Function

An intriguing clue about gH/gL function in general came from studies carried out by Vanarsdall *et al*. [44] for fusion induced by CMV glycoproteins. They found that cells could be transduced separately with Adenovirus vectors for gH/gL and gB and that fusion occurred when the two cell populations were mixed. Their result was the first description of a multi-component viral fusion machine that could be split between cells (fusion *in trans*). This startling result, which was observed prior to solution of the structure of gH/gL, was difficult to explain if gH/gL had to be in the same membrane as gB in order to act as a “co-fusogen” or even as an independent fusogen that added to a poorly fusogenic gB. Although the observation about CMV glycoproteins was met with initial skepticism (including ours), it made much more sense once the structure of gH/gL was solved. Since gH/gL did not look like a fusion protein, we first assumed that it wasn’t. In that case, we hypothesized that its ability to participate in fusion would not require it to be in the same membrane as gB. In support, we found that receptor-positive B78H1 (C10) mouse melanoma cells expressing gH/gL fused with receptor-negative B78H1 cells expressing gB and gD (fusion *in* *trans*) [184]. Second, fusion also occurred when gH/gL-expressing C10 cells that had been pre-exposed to soluble gD306 were subsequently co-cultured with gB-expressing B78H1 (receptor negative) cells. In contrast, prior exposure of gB-expressing C10 cells to soluble gD did not promote subsequent fusion with gH/gL-expressing B78H1 cells. These data support the original observation made by Vanarsdall *et al*. for CMV [44]. 

Of greater importance, current data also suggest that HSV fusion first involves activation of gH/gL by receptor-bound gD and that gH/gL does not even have to be membrane bound to function. Specifically, we found that soluble gH/gL, where gH is truncated prior to the transmembrane region, triggers a low level of fusion of C10 cells expressing gD and gB; a much higher level is achieved when gB-expressing C10 cells are exposed to a combination of soluble gH/gL and soluble gD. Of course the level of fusion induced by the soluble proteins or even when gB and gH/gL are in *trans* is much lower than what occurs when all 4 glycoproteins are *in* *cis*. This is to be expected as this is the optimal configuration, *i.e.*, that found in the virus. Fusion *in trans* and with soluble gH/gL does not preclude the possibility that the C-terminal domain of gH plays a role in the fusion process itself. The low efficiency of fusion *in trans* and other more technical issues might account for two previous reports that fusion by HSV glycoproteins was induced only when gD, gB, and gH/gL were *in cis* [185,186]. However, these experiments show that the only protein that needs to be membrane bound to function is gB and we believe this is solid evidence that it is the sole fusogen of HSV and likely to be the key fusogen of all herpesviruses.. 

## 12. Model for Herpesvirus Fusion and How It Relates to Fusion by Other Viruses

**Figure 5:**
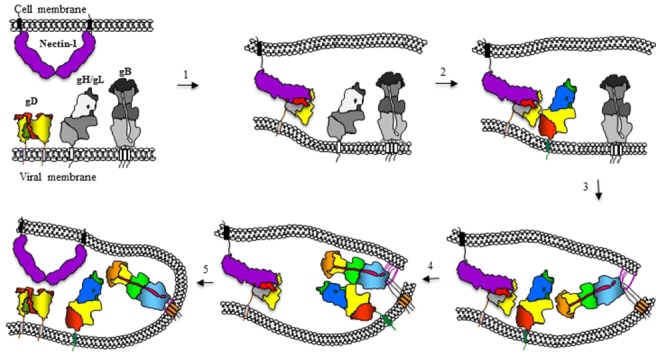
Cartoon representation of the sequential events leading to HSV entry. The entry process begins with the binding of gD to specific receptors (nectin-1 is shown), to gain entry into target cells (step 1). Receptor binding triggers displacement of the C-terminus of gD to expose a previously hidden region of gD, which we propose interacts with gH/gL (step 2). This interaction results in a conformational change in gH/gL that enables it to up-regulate gB into a fusogenic state. This three-step process may involve the interaction of gB with a cell surface protein, the insertion of gB fusion loops into the opposing lipid membrane (step 3) and an interaction between the ectodomains of gB and gH/gL (step 4). This converts gB from a pre- to a post-fusion state, resulting in fusion of the viral envelope with cell membranes and delivery of the nucleocapsid into the target cell (step 5). All essential proteins shown were drawn based on published structures with the corresponding domains. Where pre-activation structures were not available, the proteins are shown in gray.

We suggest that fusion occurs in an exquisitely regulated stepwise process (Figure 5 shows this for HSV) that consists of the following steps: (1) For HSV, gD binds to a receptor, either HVEM or nectin-1, and undergoes conformational changes in that process; for EBV, gp42 binds to HLA receptor molecule on B cells and may undergo somewhat more modest changes; (2) The activated form of these receptor-binding proteins now allows them to bind to convert gH/gL into a form that interacts with gB; this brings the two membranes together followed by insertion of fusion loops into the cell membrane (steps 3 and 4). This leads to step (5) that consists of fusion of the two membranes and delivery of the capsid into the cell (entry). We suggest that the final two steps are likely be common to other herpesviruses since they all use both gB and gH/gL for fusion. Although our model predicts that conversion of gB from a resting state to an active fusogenic state is doubly regulated in HSV and possibly EBV, we do not yet know if this mechanism for gB activation applies across the herpesviruses. However, it is especially clear that gB is the only fusion protein of HSV. 

Our data suggests that the interaction between gD and gH/gL in cells is necessarily transient or weak. Stabilization of a gD-gH/gL complex occurred even when gB was absent, excluding the possibility of fusion [96]. However, when gB was present, no fusion occurred, suggesting that this complex has to dissociate for the fusion process to proceed. When we stabilized the complex of these proteins at the C-termini of the two proteins with EYFP tags, no fusion occurred when gB and the receptor were also present [96,136]. However, there have been studies showing that gD and gH/gL can be co-precipitated from cell extracts, though it is uncertain whether this complex represents a functional one [97]. In contrast, the interaction between gp42 and gH/gL of EBV is necessarily quite strong and has a positive effect on fusion [16,55,60,187,188]. Thus, there is likely to be variability in how each of these proteins activates gH/gL; the low pH of the endosome may play an important role in accounting for these differences. It is important to note that the system we used to study gB and gH/gL *in* *cis* vs. *trans* in cell-cell fusion more closely resembles cell-cell spread than virus entry. A future challenge will be to determine how the outlines of this fusion “pathway” apply to herpesvirus entry.

Finally, we suggest that that fusion induced by herpesvirus glycoproteins is similar in at least some respects to fusion induced by the single glycoproteins of RNA viruses, such as influenza HA, flavivirus E and even VSV G [123,189]. But the closest example to herpesvirus fusion/entry is that of the paramyxoviruses which employ one viral glycoprotein to bind receptor (G) and a second virally encoded glycoprotein (F) to carry out fusion [190,191,192]. In this family of viruses, all have similar mechanisms to activate F including protease cleavage, but receptor binding varies widely [191]. Although common mechanisms of fusion protein proteolytic activation and the mechanism of membrane fusion promotion have been shown in recent years, considerable diversity exists in the family relating to receptor binding and the potential mechanisms of fusion triggering. One particularly interesting example is the *Henipavirus* genus of viruses [192,193]. Unlike many members of the Paramyxovirus family that use glycan receptors, members of the *Henipavirus* genus use protein-based receptors (ephrinB2 and ephrinB3) for virus entry [194]. Receptor binding by G triggers a cascade of conformational changes and protease cleavages that ultimately result in membrane fusion. We suggest that fusion by this group of viruses also occurs in a stepwise manner that resembles our model for how HSV fusion is accomplished (Figure 5). However, in both cases, dissecting the mechanism by which receptor binding activates the receptor binding protein which in turn activates the fusion machinery remains a challenge for future investigation. Still, structural biology coupled with biochemistry and immunology, have clarified many aspects of herpesvirus fusion and have proven that fusion is due to a single glycoprotein whose activity is exquisitely regulated by other viral glycoproteins and their receptors. 
